# An experimental study and mathematical formulation for hydrogen diffusion in water

**DOI:** 10.1038/s41598-025-28427-2

**Published:** 2025-12-29

**Authors:** Sadegh Ahmadpour, Raoof Gholami, Mojtaba Ghaedi, Martin J. Blunt

**Affiliations:** 1 Department of Energy and Petroleum Engineering, University of Stavanger, Stavanger, Norway; 2 Department of Earth Science and Engineering, Imperial College London, London, UK

**Keywords:** Hydrogen, Diffusion, Pressure decay, Temperature effects, Saline water, Chemistry, Energy science and technology, Environmental sciences, Solid Earth sciences

## Abstract

The transition to a hydrogen-based energy system requires safe and efficient large-scale storage solutions. Underground hydrogen storage (UHS) has emerged as a promising candidate, but the effectiveness and long-term security of UHS depend on a fundamental understanding of hydrogen transport mechanisms in subsurface environments. Among these, hydrogen diffusion into formation water plays a key role in governing mass loss, geochemical reactions, and microbial activity. This study presents a combined experimental and mathematical approach to quantify hydrogen diffusion in distilled water and brine at temperatures of 30 °C, 45 °C, and 60 °C, and pressures near 6.5 MPa. A new formulation is proposed that accounts for non-constant gas compressibility and enables systematic identification of the initial non-diffusive regime in pressure-time data. The methodology was validated by measuring the diffusion coefficient of CO₂ in distilled water, giving values consistent with those reported in the literature. Experimental results showed that hydrogen diffusion coefficients increase with temperature, from 3.6$\:\pm\:$0.4 × 10⁻⁹ m²/s at 30 °C to 6.1$\:\pm\:$0.3 × 10⁻⁹ m²/s at 60 °C. An additional series of tests on brine demonstrated that increasing salinity from 0 to 10,000 ppm of NaCl salt reduces the hydrogen diffusion coefficient to 5.$\:8\pm\:$0.2 × 10⁻⁹ m²/s at 60 °C. This methodology offers a robust framework for characterizing gas diffusion in aqueous systems, providing critical input not only for modeling hydrogen behavior in geological storage formations but also for optimizing hydrogen production via water electrolysis and its utilization in fuel cells.

**Supplementary Information:**

The online version contains supplementary material available at 10.1038/s41598-025-28427-2.

## Introduction

The global shift from fossil fuels to sustainable energy systems has heightened the need for alternative energy carriers that are both clean and efficient. Among these, hydrogen has emerged as a promising candidate due to its high energy density on a mass basis and the absence of greenhouse gas (GHG) emissions upon use. However, the low volumetric energy density of hydrogen necessitates the development of large-scale, seasonal storage strategies to accommodate fluctuations in supply and demand across the hydrogen value chain. Underground hydrogen storage (UHS), particularly in salt caverns, depleted hydrocarbon reservoirs, and saline aquifers, has gained attention as a viable and scalable solution. Despite its potential, several technical challenges remain before UHS can be widely implemented, including understanding and mitigating hydrogen loss mechanisms^1^.

One of the primary loss mechanisms in UHS systems is the diffusion of hydrogen into the formation water. This not only leads to mass loss but also increases the availability of hydrogen for geochemical reactions and microbial activity, which may alter reservoir integrity or generate undesirable byproducts such as hydrogen sulfide (H₂S). While hydrogen solubility in water has been well-documented in the literature^2–5^, few studies have focused on hydrogen diffusion, especially under subsurface-relevant conditions, even though this will control the rate at which the hydrogen can dissolve.

Several techniques exist to measure gas diffusion in liquids, including concentration measurements, pressure decay tests, constant pressure tests, pendant drop volume/shape analysis, nuclear magnetic resonance (NMR), X-ray computed tomography (CT) methods, laminar liquid jet absorber (LLJ), and Taylor dispersion apparatus (TDA)^6–10^. Among these, the pressure decay method is one of the most reliable approaches for quantifying gas diffusion in static liquid environments. In this method, a known volume of gas is injected into a constant-volume, liquid-filled PVT cell at a stable temperature. The subsequent pressure decline over time is then analyzed to determine the diffusion coefficient^11^.

There have been several studies where the pressure decay test was used to determine the diffusion coefficient of CO_2_ and hydrogen in water. For Instance, Zhang et al.^12^ evaluated the diffusion coefficient of CO_2_ in brine in a temperature range of 13–30 °C and a pressure of 5 MPa. To calculate the diffusion coefficient, they assumed equilibrium and infinite boundary conditions and a constant gas compressibility: the data were plotted as the pressure difference versus the square root of time. Ahmadi et al.^13^ performed a series of pressure decay experiments to determine the CO_2_ diffusion coefficient in water in the temperature range of 25–80 °C and a pressure of up to 18.5 MPa. They observed a rapid pressure drop at the beginning of their tests, due to convection that was manually corrected by truncation of the early-time data. Wang and Hou^14^ used an equilibrium boundary condition and an analytical solution to calculate the diffusion coefficient of CO_2_ in pure water and brine at 50 MPa and 120 °C. They observed a small perturbation at the beginning of the test, which was ignored when calculating the diffusion coefficient. Basilio et al.^15^ designed an improved pressure decay test to remove the effect of convection and measure the diffusion coefficient of CO_2_ in water. They manually truncated the early time to remove the perturbation region and determined the diffusion coefficient using an analytical formula. Khajooie et al.^16^ performed a similar pressure decay test and used an analytical solution where the difference of gas density was plotted against $\:ln\left(t\right)$ to calculate the diffusion coefficients of H_2_, CO_2_, He, Ar, and CH_4_ in deionized water. They were not able to get a good match with the literature for the diffusion coefficient of CO_2_ and Ar. Despite the abundance of these studies, the values reported in literature for diffusion coefficients —especially for CO₂ and H₂—vary by as much as 70%^8,16–18^, largely due to the absence of a unified and systematic framework for the correction of transient effects, the consideration of gas non-idealities, and the consistent determination of the analysis window.

This study addresses these gaps by introducing a new mathematical formulation to determine the hydrogen diffusion coefficient in distilled water and brine under low to moderate pressure and temperature conditions. The method explicitly accounts for non-constant gas compressibility and systematically isolates the semi-infinite diffusion regime from the perturbation region in the pressure-time data. By validating the approach against well-characterized CO₂ diffusion data and applying it to hydrogen at different temperatures and salinities, this work establishes a reproducible and physically grounded methodology that enhances the accuracy and reliability of gas diffusion measurements in water.

## Pressure decay test

**Fig. 1:**
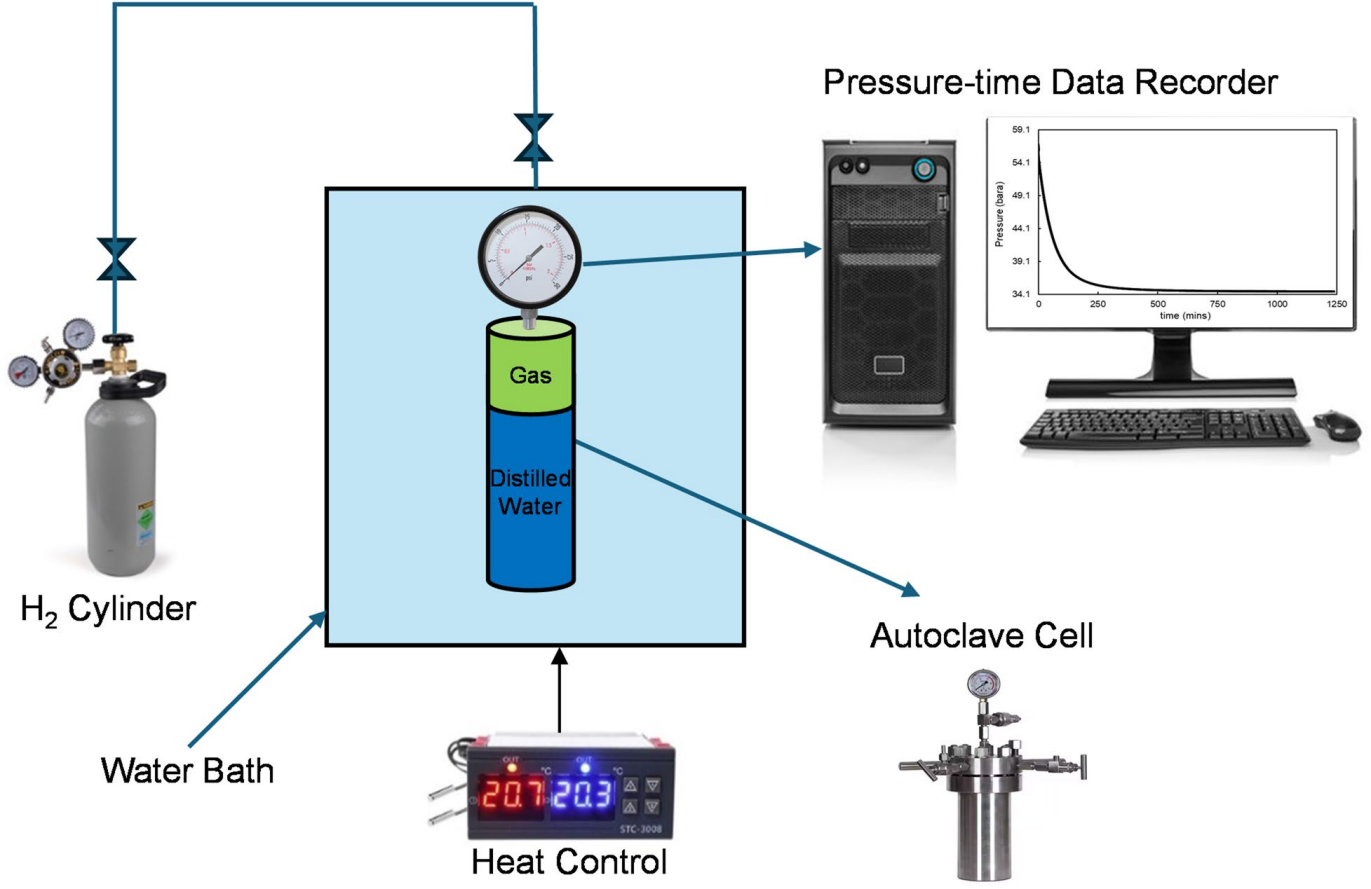
Schematic of the experimental apparatus used in this study.

To determine the diffusion coefficient of hydrogen in distilled water and brine, a series of pressure decay tests was performed using a custom-designed laboratory apparatus. The pressure decay approach was selected due to its simplicity, reliability, and ability to directly track gas mass transfer into liquids under controlled conditions. The experimental configuration is illustrated in Fig. 1.

As shown in Fig. 1, the apparatus consisted of a stainless-steel autoclave (PVT cell with an internal diameter of 52 mm and a height of 73 mm) partially filled with distilled water or brine, connected to a high-purity hydrogen gas tank. The autoclave was immersed in a thermostatically controlled water bath to maintain constant temperature throughout the duration of the test. Prior to conducting the actual diffusion tests, the integrity of the experimental system was rigorously evaluated to ensure that pressure changes would solely reflect diffusion into the liquid and not gas leakage.

### Leak testing

**Fig. 2:**
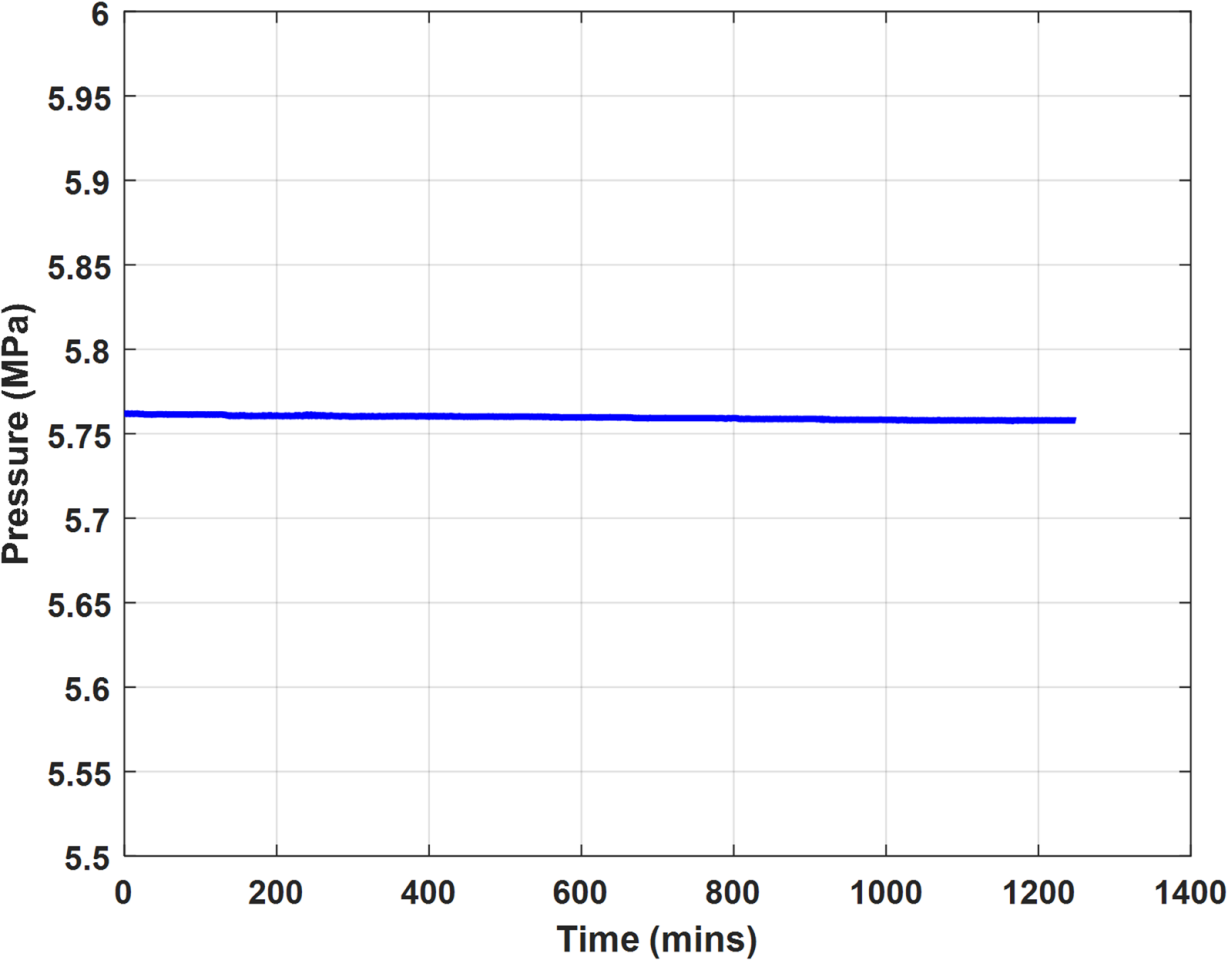
Pressure profile for the hydrogen leakage test of the cell at 30 °C.

To evaluate the integrity of the cell, the cleaned and vacuumed cell was first filled with hydrogen gas to approximately 5.75 MPa —half of its nominal pressure capacity. The system was then isolated and left undisturbed in the water bath at 30 °C for a period of over 20 h. Pressure readings were continuously monitored during this time. As shown in Fig. 2, the maximum pressure drop recorded was only 4.5 kPa over 1250 min, indicating a negligible leak rate. This minor leak correction was later incorporated into the analysis of diffusion experiments to ensure data accuracy. The procedure included calculating the pressure drop, fitting a linear line to the pressure drop curve, and compensating for the lost pressure after each test.

### Procedure for diffusion testing

After confirming system tightness, the autoclave was prepared for the hydrogen diffusion experiments. The procedure was as follows:

i. The autoclave was thoroughly cleaned and dried before weighing. High-purity distilled water (with a resistivity of 182 kΩ·m at 21.8 °C according to the manufacturer’s specification on the device) or brine was then added to fill approximately 60% of the total volume. The autoclave was weighed again to determine the precise mass and, subsequently, the volume of the liquid phase using water density.
ii. The autoclave containing the liquid was placed in the thermostatically controlled water bath for at least 24 h to ensure thermal equilibrium at the target temperature (30 °C, 45 °C, or 60 °C, depending on the test case). This step ensured that both the liquid and the gas would experience minimal thermal fluctuations during the test, which could otherwise skew pressure readings.
iii. Once thermal stability was confirmed, the autoclave was evacuated using a vacuum pump for three hours to remove any residual gases from the gas phase. The gas head of the cell mainly consisted of air and water vapor after the system reached the experimental temperature. Following degassing, hydrogen gas was injected into the autoclave to reach an initial pressure of around 6.0-7.5 MPa.
iv. After gas injection, the system was isolated, and pressure decay was continuously recorded for durations ranging from 20 to 150 h, depending on the temperature and gas-liquid system being tested. The pressure drops over time reflect the diffusion of hydrogen from the gas phase into the liquid phase across the gas-liquid interface.

This procedure was repeated under different temperatures and salinity conditions to evaluate the effect of thermal energy and ionic concentration on the hydrogen diffusion coefficient. A detailed description of the mathematical model used to interpret the pressure data and extract the diffusion coefficient is provided in the following section. The information on CO_2_ and H_2_ injected in the brine is mentioned in Table 1.

**Table 1:** Information on CO_2_ and H_2_ injected in the cell.

Chemical name	CAS number	Source	Purity (%)	T_c_ (°C)	*P*_c_ (MPa)
CO_2_	124-38-9	BOC Ltd	99.99	31	7.383
H_2_	1333-74-0	BOC Ltd	99.99	– 239.96	1.313

## Determination of the diffusion coefficient

### Mathematical model

**Fig. 3:**
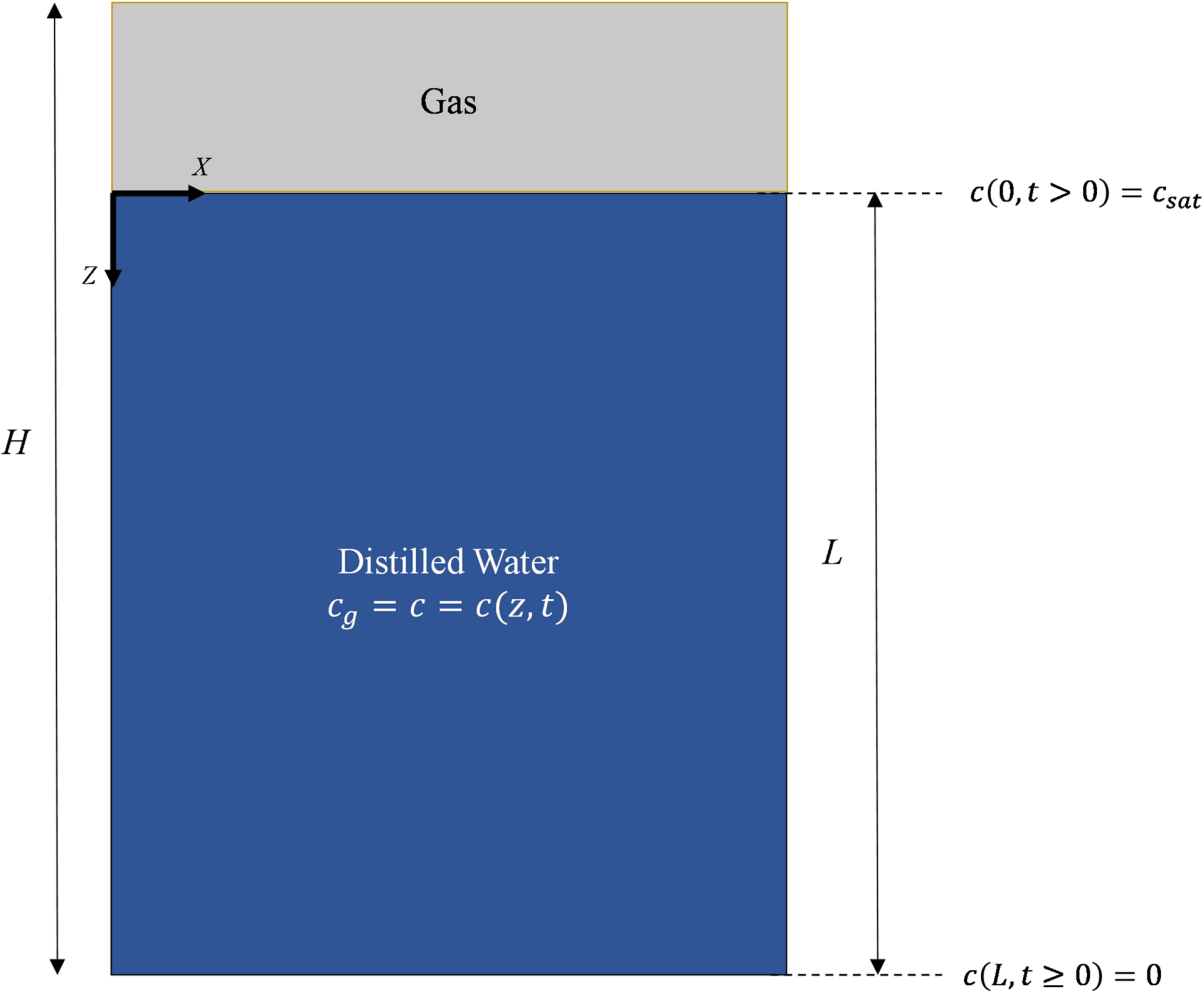
System configuration for what is assumed to be a semi-infinite flow region.

To use the pressure decay method and accurately determine the diffusion coefficient, a new mathematical formulation was developed that could be adapted to the experimental procedure and conditions described earlier. Figure 3 shows the system configuration and the boundary conditions, where the liquid height and total height are shown by $\:L$ and $\:H$, respectively.

When the system’s diameter is small relative to its height, diffusion is effectively one-dimensional along the vertical axis. Assuming that the diffusion coefficient is constant in time and space, at constant temperature, without a concentration gradient of H_2_ or any other gas component in the gas phase and without induced or natural convection in the solution, Fick’s second law can be written as follows^19^:

1 $$\:\frac{\partial\:c}{\partial\:t}=D\frac{{\partial\:}^{2}c}{\partial\:{z}^{2}}\quad 0\le\:z\le\:L,\:\mathrm{a}\mathrm{n}\mathrm{d}\:t\ge\:0$$

where $\:c=c(z,t)$. The diffusion in this system begins at $\:z=0$ and goes up to $\:z=L$ where the concentration at $\:z=0$ is constant, reaching $\:{c}_{sat}$, instantly. Here, $\:{c}_{sat}$ is the maximum solubility of the gas in the liquid at a constant temperature and the highest pressure of the system. The Initial and boundary conditions can then be defined as:

2 $$\:c\left(z,0\right)=0\quad \mathrm{f}\mathrm{o}\mathrm{r}\:0\le\:z\le\:L$$

3 $$\:c\left(0,t\right)={c}_{sat}\quad \mathrm{f}\mathrm{o}\mathrm{r}\:t>0$$

4 $$\:c\left(+\infty\:,t\right)=0\quad \mathrm{f}\mathrm{o}\mathrm{r}\:t\ge\:0$$

Solving Eq. (1) with the above boundary conditions (Eqs. 2, 3 & 4) is possible by introducing a new nondimensional variable (w), which is defined as:

5 $$\:w=\frac{z}{2\sqrt{Dt}}$$

The 1st derivative of $\:c(z,t)$ with respect to $\:t$, and the 1st and 2nd derivative of $\:c(z,t)$ with respect to $\:z$ can then be written as a function of$\:\:{c}^{{\prime\:}}=\frac{dc}{dw}$:

6 $$\:\frac{\partial\:c}{\partial\:t}=-\frac{z}{4t\sqrt{Dt}}.c^{\prime\:}$$

7 $$\:\frac{\partial\:c}{\partial\:z}=\frac{1}{2\sqrt{Dt}}.c^{\prime\:}$$

8 $$\:\frac{{\partial\:}^{2}c}{\partial\:{z}^{2}}=\frac{1}{4Dt}.{c}^{{\prime\:}{\prime\:}}$$

Inserting Eqs. (6) and (8) into Eq. (1) gives Eq. (9) with a new set of Initial and boundary conditions as below:

9 $$\:{c}^{{\prime\:}{\prime\:}}=-2w{c}^{{\prime\:}}$$

10 $$\:c\left(+\infty\:\right)=0$$

11 $$\:\:c\left(0\right)={c}_{sat}$$

12 $$\:c\left(+\infty\:\right)=0$$

Equation (9) can be solved with respect to the new boundary conditions and written as:

13 $$\:c\left(w\right)={c}_{sat}(1-{erf}\left(w\right))$$

where $\:\mathrm{erf}\left(w\right)$ is the value of the error function at $\:w$ defined as:

14 $$\:\mathrm{erf}\left(w\right)=\frac{2}{\sqrt{\pi\:}}{\int\:}_{0}^{w}{e}^{-{t}^{2}}dt$$

Equation (1) can now be written as a function of $\:t$ and $\:z$ as:

15 $$\:c\left(z,t\right)={c}_{sat}\left(1-{erf}\left(\frac{z}{2\sqrt{Dt}}\right)\right)$$

To obtain the distance from the boundary of gas and liquid as a function of gas concentration in the liquid phase (z), Eq. (13) can be rearranged as:

16 $$\:\frac{c\left(w\right)}{{c}_{sat}}=1-{erf}\left(w\right)=erfc\left(w\right)$$

17 $$\:w={erfc}^{-1}\left(\frac{c}{{c}_{sat}}\right)$$

Substituting Eq. (5) into Eq. (17) gives:

18 $$\:z=2\sqrt{Dt}\:.\:{erfc}^{-1}\left(\frac{c}{{c}_{sat}}\right)$$

By integrating both sides of Eq. (18) from $\:{c}_{sat}$ to $\:+\infty\:$, and multiplying them by the cross-sectional area of the gas-liquid ($\:A$), the number of moles of diffused gas component in the liquid phase at each time is obtained as:

19 $$\:{n}_{d}\left(t\right)=A{\int\:}_{0}^{{c}_{sat}}z\left(c\right)dc=2A\sqrt{Dt}{\int\:}_{0}^{{c}_{sat}}{erfc}^{-1}\left(\frac{c}{{c}_{sat}}\right)dc$$

Assuming that $\:q=\frac{c}{{c}_{sat}}$, Eq. (19) can be written as:

20 $$\:{n}_{d}\left(t\right)=2A{c}_{sat}\sqrt{Dt}{\int\:}_{0}^{1}{erfc}^{-1}\left(q\right)dq$$

where $\:{\int\:}_{0}^{1}{erfc}^{-1}\left(q\right)dq$ is constant and equal to $\:\frac{1}{\sqrt{\boldsymbol{\pi\:}}}$. Therefore, Eq. (20) can be written as:

21 $$\:{n}_{d}\left(t\right)=\frac{2}{\sqrt{\boldsymbol{\pi\:}}}A{c}_{sat}\sqrt{Dt}$$

As gas molecules diffuse into the liquid phase, the pressure in the gas phase decreases. Consequently, $\:{n}_{d}\left(t\right)$ in Eq. (21) will be proportional to the gas pressure as a function of $\:\sqrt{t}$​ if the ideal gas law is assumed, later we show how to relax this assumption.

**Fig. 4:**
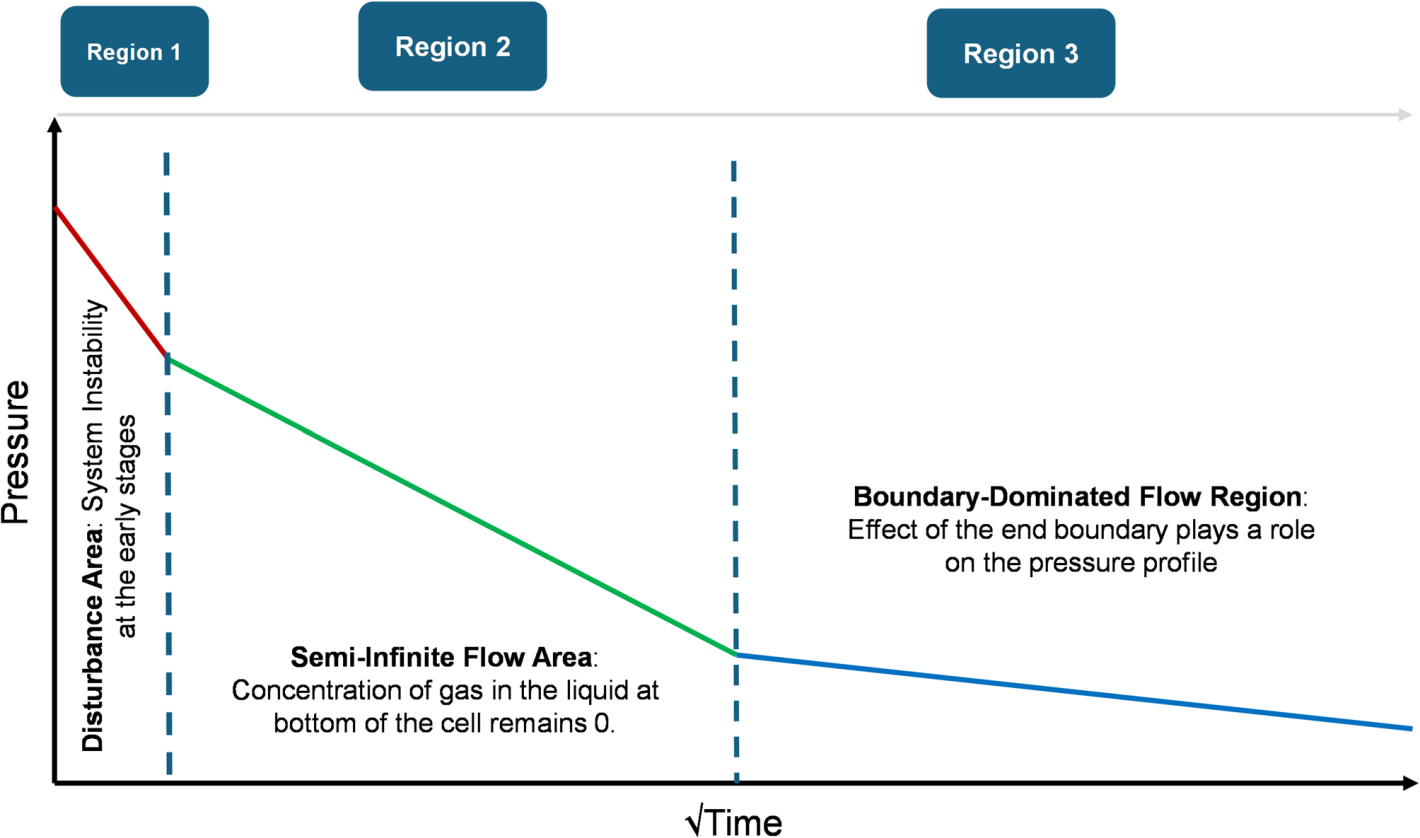
Suitable time window identification for the diffusion coefficient determination according to the pressure decay test data.

As illustrated in Fig. 4, the pressure decay can be divided into three characteristic regions once the pressure change is plotted against the square root of time. The first region, referred to as the perturbation region, represents the early-time response immediately after gas injection. This period is dominated by non-diffusive effects such as pressure stabilization, thermal equilibration, and possible turbulence at the gas-liquid interface. Its duration is influenced by several factors, including injection rate, target pressure, temperature, gas properties, and cell geometry. The second region is the semi-infinite diffusion regime, where the boundary at the base of the liquid can be considered sufficiently distant such that it does not affect diffusion behavior. This region aligns with the assumptions used in the analytical solution and is characterized by a linear relationship between pressure drop and the square root of time. Accurate estimation of the diffusion coefficient relies on selecting data exclusively from this regime. Finally, the third region, termed the boundary-dominated regime, begins when the diffusing front approaches the bottom of the liquid column. In this phase, the rate of pressure drop slows and deviates from the earlier linear trend. Identifying these regions in the pressure decay profile is crucial for isolating the valid time window over which the analytical model can be applied reliably to extract diffusion coefficients.

### Mathematical model to find the diffusion coefficient

To account for non-ideal gas properties, it was assumed that the gas phase obeys the real gas law, *PV*_*g*_ *= Z*_*g*_*nRT*, where the compressibility correction factor, *Z*_*g*_, is a function of gas composition, temperature, and pressure. It was also assumed that the diffusion of gas from the gas phase into the liquid phase at their interface follows Fick’s 1st law^19^. Then the change in the amount of gas phase is equal to the amount of gas entering the liquid phase from the interface, which can be expressed as:

22 $$\:{dn}_{gas\:phase}={dn}_{at\:the\:interface}\to\:\frac{{V}_{g}}{{Z}_{g}RT}dP={j}_{|z=0}Adt=-DA{\left(\frac{\partial\:c}{\partial\:z}\right)}_{|z=0}dt$$

To obtain $\:\frac{\partial\:c}{\partial\:z}$, the 1st derivative of Eq. (15) at $\:z=0$ can be considered and written as:

23 $$\:{\left(\frac{\partial\:c}{\partial\:z}\right)}_{|z=0}=-\frac{{c}_{sat}}{\sqrt{\pi\:Dt}}$$

Alternatively, $\:{\left(\frac{\partial\:c}{\partial\:z}\right)}_{|z=0}$ can be obtained using Eq. (21), where $\:{dn}_{gas\:phase}$ can be obtained as:

24 $$\:{dn}_{gas\:phase}\left(t\right)=\frac{2}{\sqrt{\boldsymbol{\pi\:}}}A{c}_{sat}\sqrt{Ddt}$$

Changes in the pressure can then be obtained by inserting Eq. (24) into Eq. (22) as:

25 $$\:\frac{1}{{Z}_{g}}dP=\sqrt{D}A.\frac{{c}_{sat}}{\sqrt{\pi\:}}.\frac{RT}{{V}_{g}}.\frac{dt}{\sqrt{t}}$$

Assuming that the movement of the gas-liquid interface is negligible over time, it can be assumed that the gas volume ($\:{V}_{g}$) is constant. Furthermore $\:{V}_{g}$ is equal to $\:A\left(H-L\right)$ based on Fig. 3. Under these conditions, Eq. (25) can be integrated with respect to pressure from $\:{P}_{i}$ to $\:P$ on the left-hand side and with respect to time from $\:0$ to $\:t$ on the right-hand side, yielding:

26 $$\:{\int\:}_{{P}_{i}}^{P}\frac{1}{{Z}_{g}}dP=-\frac{2}{\sqrt{\pi\:}}.\frac{{c}_{sat}RT\sqrt{D}}{\left(H-L\right)}.\left(\sqrt{t}\right)$$

This is the exact analytical solution for the pressure decay test that is obtained with the assumption of constant $\:D$ over time.

As previously mentioned, during the initial stages of the diffusion cell experiment, non-diffusive effects influence the pressure behavior. These early-time deviations cannot be modelled using the proposed formulation. Therefore, the presented model is only applicable after the initial perturbation period, specifically, from the onset of the semi-infinite flow regime. Accordingly, Eq. (26) can be rewritten to reflect the experimental conditions, as compared to the theoretical model:

27 $$\:{\int\:}_{{P}_{int,semi-inf}}^{P}\frac{1}{{Z}_{g}}dP=-\frac{2}{\sqrt{\pi\:}}.\frac{{c}_{sat}RT\sqrt{D}}{\left(H-L\right)}.(\sqrt{t}-\sqrt{{t}_{0}})$$

Here, $\:{P}_{int,semi-inf}$ represents the pressure at the onset of the semi-infinite regime ($\:{t}_{0}$) under experimental conditions. A plot of $\:{\int\:}_{{P}_{i}}^{P}\frac{1}{{Z}_{g}}dP$ versus $\:\sqrt{t}$ will then theoretically give a linear line with a slope of $\:m=-\frac{2}{\sqrt{\pi\:}}.\frac{{c}_{i}RT}{\left(H-L\right)}.\sqrt{D}$ from which the diffusion coefficient is estimated as:

28 $$\:D=\frac{\pi\:}{4}.{\left(\frac{H-L}{{c}_{sat}RT}\right)}^{2}.{m}^{2}$$

and the intercept is $\:b=-m.\sqrt{{t}_{0}}$. If the pressure change in the diffusion cell is not significant, an approximate solution can be obtained by using an average value of the gas compressibility factor, $\:\stackrel{-}{{Z}_{g}}$, over the pressure range from $\:{P}_{i}$ to $\:P$. Accordingly, the approximate solution is:

29 $$\:{P}_{i}-P=-\frac{2}{\sqrt{\pi\:}}.\frac{{c}_{sat}RT\stackrel{-}{{Z}_{g}}\sqrt{D}}{\left(H-L\right)}.(\sqrt{t}-\sqrt{{t}_{0}})$$

Naturally, since this formulation assumes semi-infinite domain behavior, it is only valid before the pressure disturbance reaches the bottom of the diffusion cell and before the onset of boundary-dominated flow.

## Validation

To validate the accuracy and reliability of the proposed mathematical formulation for estimating hydrogen diffusion coefficients using the pressure decay method, a series of benchmark experiments was conducted using carbon dioxide (CO₂). CO₂ was chosen because its diffusion in water has been extensively studied, and a range of experimentally derived values are available in the literature^14,15,20–24^ under well-characterized thermodynamic conditions.

The validation experiments were performed under conditions similar to those used for the hydrogen diffusion tests. Distilled water was used as the liquid medium, and the test was conducted at a temperature of 30 °C and a pressure of approximately 5.3 MPa. The pressure decay was recorded over time, and the dataset was analyzed using the same mathematical approach developed in this study, which takes into account the non-constant gas compressibility and the accurate identification of the semi-infinite diffusion regime. The supplementary material provides the actual pressure profile for this case.

**Fig. 5:**
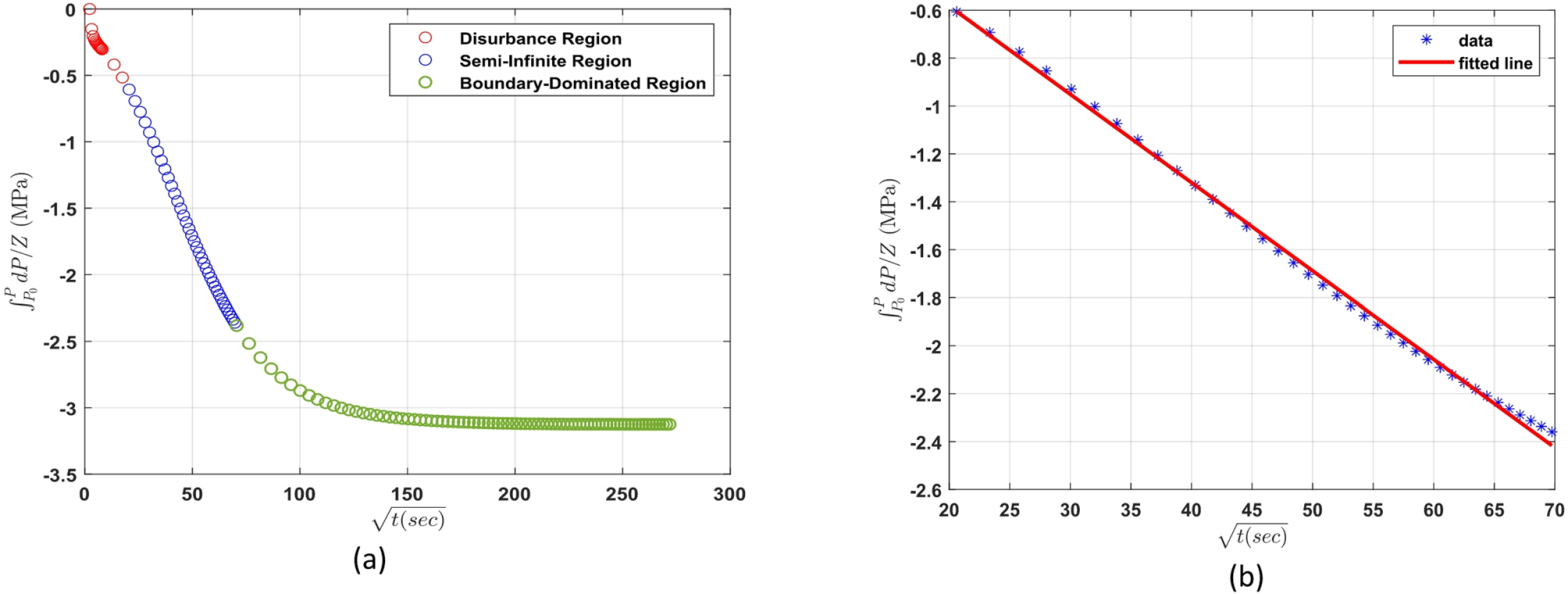
(**a**) Pressure profile versus square root of time and (**b**) fitted line used to calculate the diffusion coefficient in the semi-infinite diffusion regime for Case 1.

As shown in Fig. 5a, the pressure-time curve exhibited three distinct regions. The initial portion of the curve (approximately the first 27 min) was identified as the perturbation region, where transient effects such as injection turbulence and thermal equilibration dominate. Following this, the system entered the semi-infinite diffusion regime, characterized by a steady decrease in pressure due solely to molecular diffusion of CO₂ into the liquid phase. This regime continued until boundary effects began to dominate, indicating the start of the third region — the boundary-dominated flow regime — at around 140 min.

To determine the diffusion coefficient, only the semi-infinite regime (from 6.5 min to 84 min) was used. In this regime, $\:{\int\:}_{{P}_{i}}^{P}\frac{1}{{Z}_{g}}dP$ was plotted against the square root of time, as shown in Fig. 5b. The linearity of this plot confirms that diffusion is the dominant transport mechanism during this period and that the mathematical model assumptions are valid. From the slope of the fitted line, the diffusion coefficient of CO₂ in distilled water at the test conditions was calculated.

To verify the reliability of the results, the measured CO₂ diffusion coefficient was compared with the values from previous experimental studies as reported in Table 2. As can be seen, the value determined in the present study falls well within the range of established literature values for comparable temperature and pressure conditions. This successful validation against CO₂ establishes confidence in the use of the proposed pressure decay analysis for other gases, particularly hydrogen. Since CO₂ is more soluble and exhibits more pronounced non-ideal gas behavior under the test conditions, the accurate estimation of its diffusion coefficient also serves to demonstrate the model’s ability to handle compressibility variations and transition regimes in pressure decay data. In addition, since the pressure difference between Case 1 and Case 2 is small, a linear relation between pressure and diffusion coefficient can be assumed. Therefore, the diffusion coefficient at an average pressure of 5.08 MPa was obtained as 4.$\:3\pm\:$0.1 × 10^−9^ m^2^/s. It should be noted that factors such as water expansion, water swelling, pressure fluctuations, unstable injection, and convective mixing at the beginning of the tests can reduce the accuracy of estimating the diffusion coefficient. For instance, a rise of 0.5 mm in water height decreases the diffusion coefficient by almost 20% and unfortunately, the actual water height in the cell could not be recorded once the system was placed in the water bath.

**Table 2:** Summary of the CO_2_ diffusion coefficient in distilled water from various literature sources.

Article	Pressure (MPa)	Temperature (°C)	Approach	D × 10^− 9^ (m^2^/s)
This Study – Case 1	5.30	30	Pressure decay	4.4
This Study – Case 2	4.86	30	Pressure decay	4.1
Azin et al.^25^	5	20	Pressure decay	6.8
Cadogan et al.^26^	1–5	25–150	Taylor dispersion	1.3–4
Ahmadi et al.^13^	1–20	25–80	Pressure decay	2–7
Sharafi et al.^27^	2–20	25–80	Pressure decay	2-7.5

## Hydrogen diffusion

### Hydrogen diffusion in distilled water

To evaluate the hydrogen diffusion in distilled water, six pressure decay experiments were conducted at temperatures of 30 °C, 45 °C, and 60 °C, while maintaining a relatively constant initial pressure of approximately 6.5 MPa. In all cases, the same volume of distilled water was added to the autoclave, ensuring that the only variable affecting the diffusion coefficient was temperature. The calculated hydrogen diffusion coefficients for each test are summarized in Table 3. The values range from 3.2 × 10⁻⁹ m²/s at 30 °C to 6.3 × 10⁻⁹ m²/s at 60 °C, demonstrating a clear and significant increase with temperature. Despite efforts to maintain constant pressure across all tests, minor fluctuations were observed. However, the relation between pressure and diffusion coefficient could still be assumed linear^10,26,28^. The values of pressure and diffusion coefficient with their standard deviation (s_D_) were reported in Table 3.

**Table 3:** Hydrogen diffusion coefficients in distilled water.

Case Number	Temperature (°C)	Pressure (MPa)	Water length (mm)	C_sat_ (gmole of H_2_/m^3^ of Solution)	D × 10^–9^(m^2^/s)	Average pressure (MPa)	($\bar{\boldsymbol{D}}$± σ_D_)× 10^–9^(m^2^/s)
1a	30	6.45	48	46.2933	4.0	6.75	3.6 ± 0.4
1b	30	7.04	48	50.9345	3.2		
2a	45	6.78	49	46.2005	5.3	6.83	4. ± 0.4
2b	45	6.87	49	46.7803	4.4		
3a	60	6.83	48	46.0916	6.3	6.93	6. ± 0.3
3b	60	7.02	48	47.3158	5.8		

**Fig. 6:**
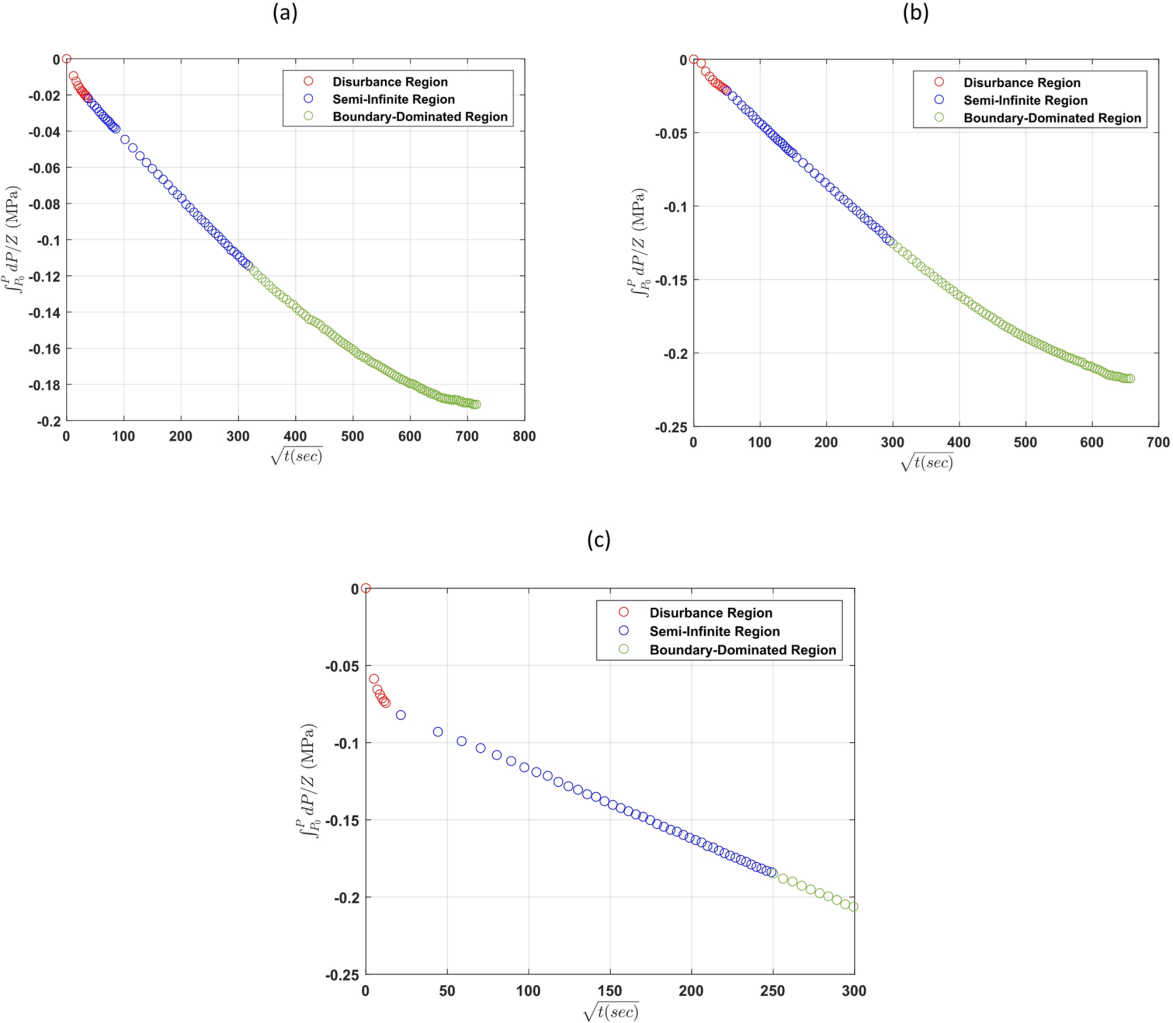
$\:{\int\:}_{{P}_{i}}^{P}\frac{1}{{Z}_{g}}dP$ profiles for the three cases used to find the hydrogen diffusion coefficient: Case 1a (**a**), Case 2a (**b**), Case 3b (**c**).

Figure 6 presents the $\:{\int\:}_{{P}_{i}}^{P}\frac{1}{{Z}_{g}}dP\:$profiles recorded during three of the tests (cases 1a, 2a, and 3b). Although the overall magnitude of pressure drop was relatively small—particularly when compared to CO₂ diffusion experiments—the pressure response still exhibited a clear and consistent downward trend over time for all of them. Importantly, the slope of the pressure decay increased with temperature for all cases, indicating a faster rate of hydrogen transfer from the gas phase into the liquid phase at elevated temperatures. It should be mentioned that the supplementary material shows the actual pressure profiles of these cases.

Following the methodology validated in “Validation”, the semi-infinite diffusion regime was carefully identified (linear relationship between $\:{\int\:}_{{P}_{i}}^{P}\frac{1}{{Z}_{g}}dP$ and square root of time) and used to find the diffusion coefficient. In this regime, $\:{\int\:}_{{P}_{i}}^{P}\frac{1}{{Z}_{g}}dP\:$was plotted against the square root of time to extract the diffusion coefficient.

**Fig. 7:**
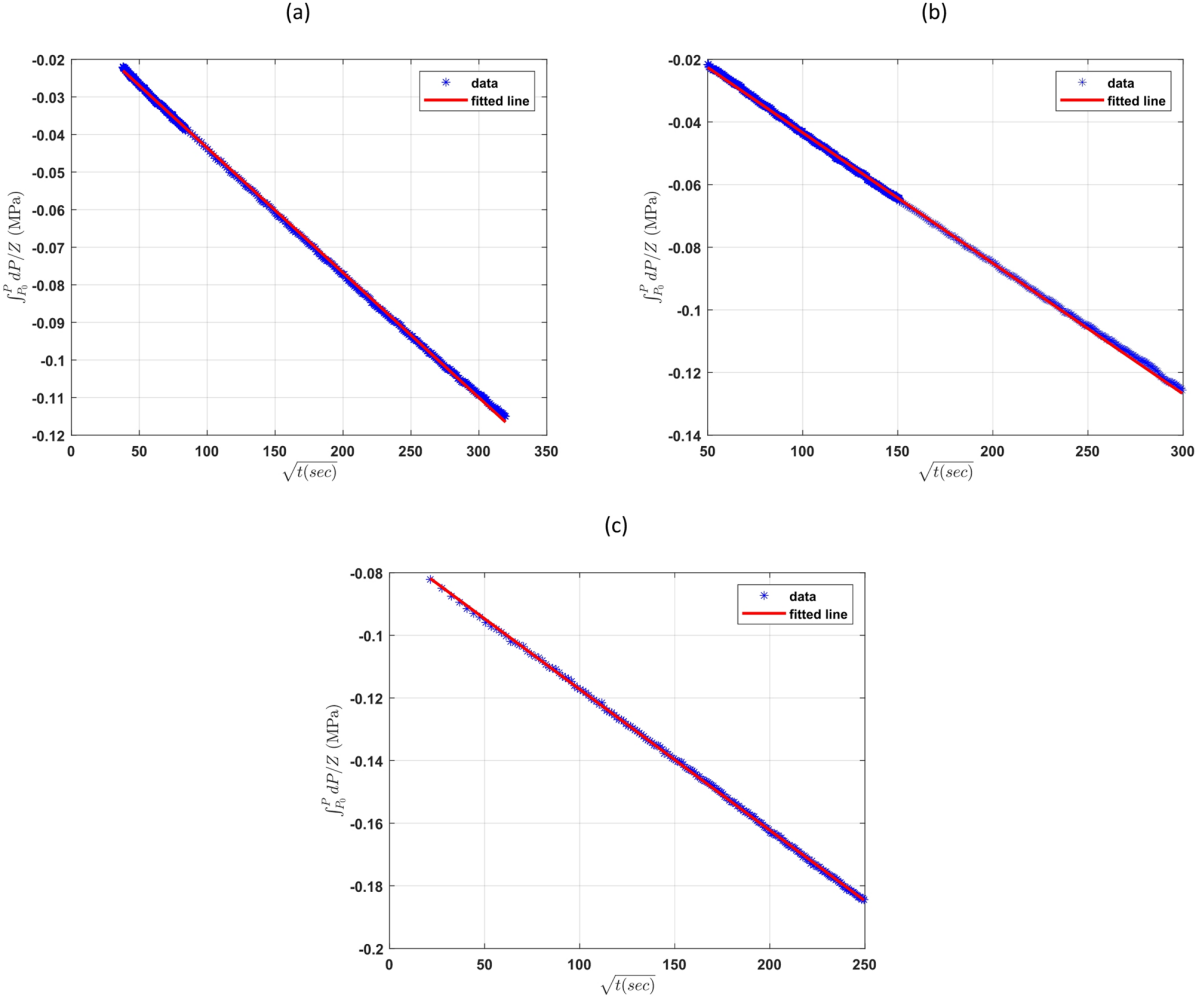
Plot of $\:{\int\:}_{{P}_{i}}^{P}\frac{dP}{{Z}_{g}}$ versus $\:\sqrt{t}$ for the 3 cases of hydrogen diffusion coefficient tests: Case 1a (**a**), Case 2a (**b**), Case 3b (**c**) in the second region and their fitted lines.

As illustrated in Fig. 7 (shown just for cases 1a, 2a, and 3b), a linear relationship was observed​, confirming that diffusion is the dominant mechanism during this phase. The slope of the fitted line increased with temperature, reflecting the enhanced mobility of hydrogen molecules at higher thermal energies. This trend aligns with established theoretical expectations and has also been reported in diffusion studies of other gases such as CO₂ and CH₄^10,21,26^.

To further validate the accuracy of the estimated diffusion coefficients, a comparative analysis was conducted with the values reported in the literature. As seen in Table 4, the values determined in this study are consistent with previously reported ranges for hydrogen diffusion in distilled water. The variation in literature values—spanning from 3 to 15 × 10⁻⁹ m²/s—can largely be attributed to differences in experimental design, temperature, pressure, and data interpretation methods. In contrast, the systematic approach used in this study, particularly the handling of the perturbation region and the non-constant gas compressibility, provides greater confidence in the derived coefficients.

**Table 4:** Summary of the H_2_ diffusion coefficient in distilled water reported in the literature.

Article	Pressure (MPa)	Temperature (°C)	Approach	D × 10^− 9^ (m^2^/s)
Wise and Houghton^29^	1	10–60	Collapse of bubble size	4.5–14
Ferrell and Himmelblau^30^	1	10–50	Laminar dispersion	3–8
De Blok and Fortuin^31^	1	20–60	Constant bubble size	3–6.5
Jähne et al.^32^	1	5–35	Time-lag	4-6.5
Tsimpanogiannis et al.^8^	0.1–200	2–702	Molecular dynamics simulations	1–50
Wang et al.^10^	4–30	5–150	Taylor dispersion	4–19
van Rooijen et al.^9^	400	25–450	Molecular dynamics simulations	2–300
Kalati et al.^33^	200	50–100	Molecular dynamics simulations	8–15
Kerkache et al.^34^	1–200	25–200	Molecular dynamics simulations	4–35
Khajooie et al.^16^	0.5	35	Pressure decay method	6.3–7

### Hydrogen diffusion in brine

To investigate the influence of salinity on hydrogen diffusion, four additional pressure decay experiments were performed using brine solutions with two differing salt concentrations. These tests were conducted at 60 °C and approximately 7 MPa to maintain consistency with the highest temperature case from the distilled water experiments, enabling a direct comparison between fresh and saline conditions.

The brine samples were prepared by dissolving sodium chloride (NaCl) into distilled water to achieve target salinities. The resulting salt concentrations were approximately 1000 ppm and 10,000 ppm, representing a low-salinity and a moderately saline system, respectively. The test conditions, including salt mass, water mass, and resulting salinity, are summarized in Table 5.

**Table 5:** Conditions of the hydrogen diffusion experiments for the brine samples.

Case	Pressure (MPa)	Temperature (°C)	NaCl (g)	Distilled water (kg)	Salinity (ppm)
1a	6.09	60	0.15	0.15017	1000
1b	7.26		0.15	0.15010	1000
2a	6.95		1.53	0.15053	10,000
2b	7.21		1.5548	0.15422	10,000

**Fig. 8:**
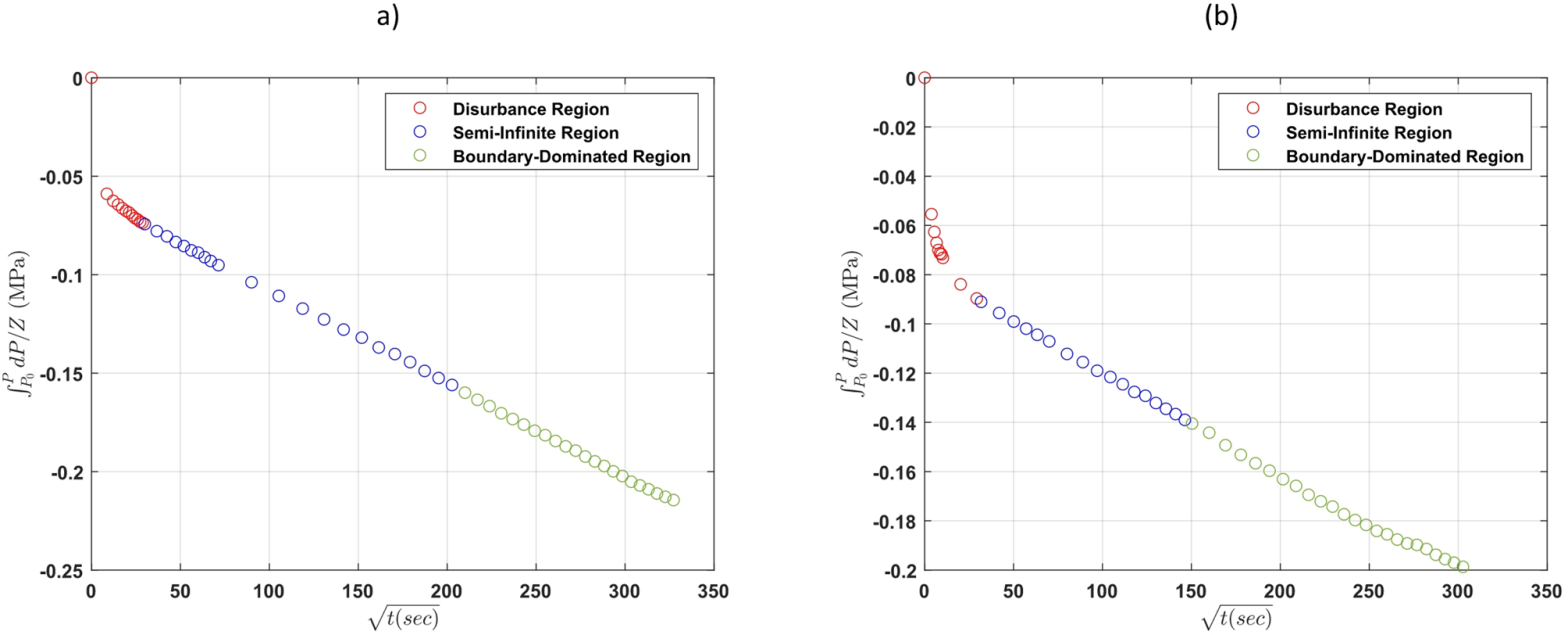
$\:{\int\:}_{{P}_{i}}^{P}\frac{1}{{Z}_{g}}dP\:$profile for the 2 cases of hydrogen diffusion in brine for Case 1b (**a**), Case 2a (**b**).

Figure 8 presents the pressure decay profiles for two of the brine cases. Similar to the distilled water tests, they show a gradual pressure decline over time, with noticeable differences in slope between the two salinity levels. The pressure drop in the low-salinity brine was more pronounced than in the high-salinity case, suggesting faster hydrogen diffusion under less saline conditions. The actual pressure profiles of these cases can be found in the supplementary material.

**Fig. 9:**
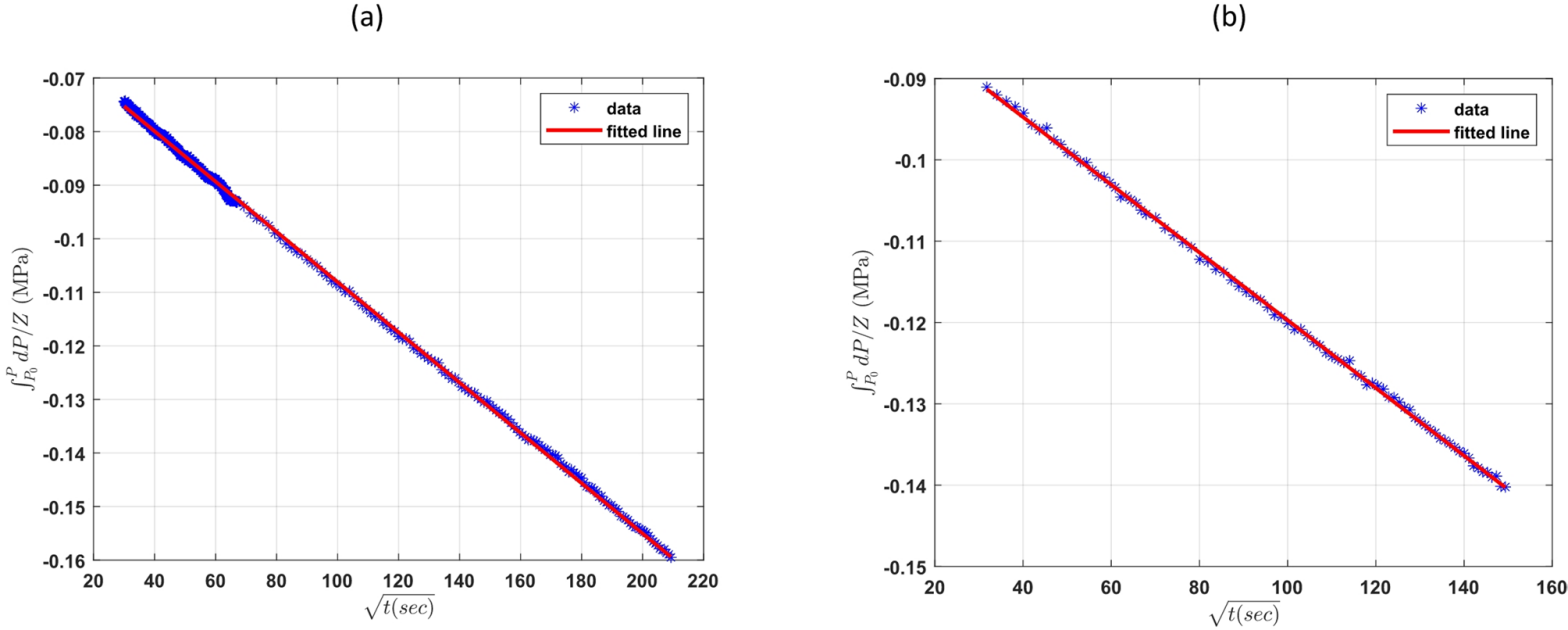
Plot of $\:{\int\:}_{{P}_{i}}^{P}\frac{dP}{{Z}_{g}}$ versus $\:\sqrt{t}$ for hydrogen diffusion in brine, Case 1b (**a**), Case 2a (**b**).

Following the same analytical procedure described in previous sections, the perturbation region was identified, and the pressure data from the semi-infinite diffusion regime were used to calculate the diffusion coefficients. As shown in Fig. 9, the pressure change was plotted against the square root of time, and linear fits were used to extract the diffusion coefficients from the slope.

The hydrogen diffusion coefficients for the four cases are presented in Table 6. The results clearly indicate a decrease in diffusion coefficient with increasing salinity, consistent with theoretical expectations and findings from previous studies. These findings align with the general understanding that increased ionic concentration reduces gas mobility in aqueous systems. The presence of dissolved ions such as Na⁺ and Cl⁻ increases the solution’s viscosity and alters its molecular structure, reducing the diffusivity of dissolved gases like hydrogen^33,34^. The lower diffusion coefficient observed in Case 1a compared to Cases 2a and 2b, despite its lower salinity, can be attributed to the lower pressure conditions in Case 1a, which led to a reduction in diffusivity. As highlighted earlier, an additional challenge was the uncertainty in the actual water height due to thermal expansion, which could not be measured. Given the relatively small variation of the diffusion coefficient with pressure, a linear correlation between pressure and diffusion coefficient was assumed, and the average values with their standard deviations are reported in Table 6.

**Table 6:** The results of the hydrogen diffusion test on the Brine samples.

Case	Pressure (MPa)	Brine length (mm)	C_sat_ (gmole of H_2_/m^3^ of Solution)	Temperature (°C)	Salinity (ppm)	$\:{\boldsymbol{D}}_{{\boldsymbol{H}}_{2}}$×10^–9^(m^2^/s)	Average pressure (MPa)	($\:\bar{\boldsymbol{D}}$ ± σ_D_)×10^–9^ (m^2^/s)
1a	6.09	48	39.4005	60	1000	4.9	–	–
1b	7.26	49	46.6052		1000	6.0		
2a	6.95	49	42.6472		10,000	5.6	7.08	5.8 ± 0.2
2b	7.21	50	44.1802		10,000	5.9		

To contextualize these results, Table 7 provides a comparison of hydrogen diffusion coefficients in brines reported in recent literature using both experimental and simulation-based techniques. Despite differences in methodology and brine composition, the diffusion coefficients measured in this study fall within the reported range of 1–15 × 10⁻⁹ m²/s, demonstrating the consistency of the results.

**Table 7:** Summary of the H_2_ diffusion coefficient in Brine reported in the literature.

Article	Pressure (MPa)	Temperature (°C)	Salinity/salt type	Approach	D × 10^− 9^ (m^2^/s)
Bhimineni et al.^35^	0.1013–22.0889	15–95	0–5 m of NaCl and KCl	Machine learning/molecular dynamics simulation	1–6
Kalati et al.^33^	0.1–20	50–100	0–5 of NaCl or MgCl_2_	Molecular dynamics simulation	2–15
Kerkache et al.^34^	0.1–20	25–200	0–6 m of NaCl	Molecular dynamics simulation	3–30
Rezk^18^	3–11	35–50	NaHCO3: 0.17 mg/L Na2SO4.10.H2O: 7.93 mg/L NaCl: 41.17 mg/L CaCl2.4.H2O: 2.97 mg/L MgCl2.6H2O: 17.42 mg/L	Pressure decay method	5.5–45
Piroozi et al.^36^	0.1–100	300–1000	0–6 m of NaCl	Machine learning/molecular dynamics simulation	0.01–300

## Discussion

The pressure decay method, as demonstrated in this study, provides a practical and robust approach for quantifying the diffusion coefficients of gases in liquids under controlled laboratory conditions. While the method is conceptually simple—monitoring pressure drop over time due to gas diffusion into a liquid—it involves several challenges that must be carefully addressed to ensure accurate interpretation of results.

One important source of uncertainty is the change in the height of the liquid column during the experiment due to liquid expansion and gas diffusion. Although this change is generally small, especially when the diffusion path is long, it can affect the geometry of the diffusion interface and, in turn, influence the calculated diffusion coefficient. Future refinements of the method could benefit from dynamic tracking or correction of the changing liquid level to further improve accuracy, particularly in long-duration tests or in systems with higher gas solubility.

Another critical aspect is the mathematical model used to interpret the pressure decay data. Different models can yield varying diffusion coefficients due to differences in underlying assumptions, such as gas compressibility behavior, boundary conditions, or treatment of early-time perturbations. Many studies in the literature neglect the early perturbation region or apply arbitrary truncation, which can lead to inconsistencies in reported values. The present work addresses this by introducing a systematic framework for identifying and removing the perturbation region, while explicitly incorporating a non-constant compressibility factor, which is particularly relevant at moderate to high pressures.

The uncertainties in estimating diffusion coefficients, which were reported in this study, have also been reported in previous studies. Khajooie et al.^16^ measured the diffusion coefficients of various gases in distilled water at a temperature and pressure of 35 °C and 1 MPa, respectively, using the pressure decay method. They found that hydrogen diffusion coefficients at the same conditions vary between 1.9 × 10⁻⁹ and 3.4 × 10⁻⁹ m²/s, representing a deviation of nearly 50%. Similarly, Etminan et al.^37^ measured the diffusion coefficients of CO₂ and CH₄ in heavy oil and bitumen under various pressures and temperatures using the constant pressure method. They observed changes of 11% and 7.6% in diffusivity, which were attributed to the pressure fluctuations and liquid swelling, respectively. These highlight the significant challenges of measuring diffusion coefficients in laboratory settings using either constant-pressure or constant-volume methods, where issues such as pressure instability, liquid expansion, and convection mixing can introduce considerable errors.

Interestingly, despite hydrogen’s higher diffusion coefficient compared to CO₂, the overall pressure drop observed during the hydrogen tests was significantly smaller. This initially counterintuitive result can be explained by several key physical phenomena:

i. **Compressibility behavior**: Under the tested conditions, CO₂ exhibits non-ideal gas behavior, with a compressibility factor (Z) varying between 0.64 and 0.79. In contrast, hydrogen behaves almost ideally in this pressure-temperature range, with almost constant Z values between 1.019 and 1.025. This means that CO₂ experiences greater changes in molar volume and pressure during dissolution, even for similar amounts of gas loss.
ii. **Solubility effects**: CO₂ has significantly higher solubility in water than hydrogen. Consequently, for the same initial pressure, more moles of CO₂ dissolve into the water, resulting in a larger pressure drop. As mentioned in previous studies^38^, this higher solubility leads to more substantial gas-phase depletion during the experiment.
iii. **Molecular size and interaction**: Hydrogen, being a small nonpolar molecule, interacts weakly with surrounding molecules and exhibits high mobility. However, its small molecular size also means that the number of moles removed per unit of dissolved mass is low. Moreover, the relatively low interaction potential results in less energy exchange and lower measurable pressure drop despite high diffusion rates.
iv. **Gas loss and test duration**: In the CO₂ experiments, approximately 46% of the initial gas moles were lost to the aqueous phase, while in the hydrogen tests, only about 2% of gas-phase hydrogen was consumed. This explains why, despite having higher diffusion coefficients, hydrogen generated a smaller pressure drop—there was simply less gas transferred.
v. **Equilibrium time**: These observations also influence the time to reach equilibrium. Due to its higher solubility and compressibility effects, CO₂ takes significantly longer to reach a steady state, extending the duration of the semi-infinite flow regime. In contrast, hydrogen, with limited dissolution and near-ideal behavior, equilibrates more rapidly, resulting in a shorter window for accurate diffusion analysis.

While the procedure utilized in this work is simple and fast for calculating the diffusion coefficient, some challenges influence the accuracy of the measurements and should be addressed. The first and most important challenge is that after filling the cell with pure water or brine and placing it in the bathtub, the temperature increases from ambient to the test temperature, causing slight evaporation of the liquid and consequently a change in liquid height. In addition, applying a vacuum to the cell may draw out a small amount of liquid, which can also alter the liquid height. Generally, a change in liquid height due to evaporation may also affect the salinity of the liquid in the case of saline water. In our calculations, the liquid height was measured before temperature stabilization and vacuuming, and it might differ from the actual value afterward. It was observed that even a 1 mm change in liquid height can alter the diffusion coefficient by approximately 20%. In future tests, directly measuring the actual liquid height after stabilization and vacuuming, as well as minimizing evaporation and ensuring proper vacuuming, can improve the accuracy of the results.

In addition, measuring the diffusion coefficient of pure H₂ in water or brine alone may not fully represent realistic underground storage conditions, as the gas phase often contains multiple components (e.g., H₂, cushion gases, and residual gases such as CH₄) and other dissolved ions may be present in water. However, to numerically estimate the effective diffusion coefficient of H₂ from such gas mixtures in brine containing various dissolved species, the binary diffusion coefficient of pure H₂ in brine is required as a fundamental parameter^39^.

The observed decrease in diffusion coefficient with increasing salinity suggests that brine-dominated formations may offer better hydrogen retention over long durations and reduce losses posed by microbial or geochemical reactions. Finally, the methodology developed and validated here—particularly the mathematical formulation with real gas effects and perturbation correction—offers a generalizable framework applicable to other gas-liquid systems beyond hydrogen, including methane, helium, and nitrogen, under subsurface conditions.

## Conclusions

This study presents a systematic experimental and analytical framework to accurately determine the diffusion coefficient of hydrogen in distilled water and brine using pressure decay tests. Recognizing the critical role of hydrogen diffusion in subsurface processes, particularly in the context of underground hydrogen storage (UHS), the methodology was designed to address limitations commonly encountered in previous studies—such as neglecting gas compressibility variations and relying on arbitrary truncation of early-time data.

A new mathematical formulation was developed that incorporates a non-constant gas compressibility factor and allows for the identification of distinct flow regimes in the pressure-time response, including the perturbation region, the semi-infinite diffusion regime, and the boundary-dominated phase. This systematic approach enhances the reliability and reproducibility of diffusion coefficient estimation. The method was validated through pressure decay tests using CO₂, yielding diffusion coefficients consistent with values reported in the literature. The main findings of this work can be mentioned as follows:

- In a pressure decay test, three distinctive regions are present, including disturbance, semi-infinite, and boundary-dominated regions.
- These three regimes can be identified by plotting pressure or $\:{\int\:}_{{P}_{i}}^{P}\frac{dP}{{Z}_{g}}$ versus $\:\sqrt{t}$.
- Application of the method to hydrogen revealed a strong temperature dependence, with diffusion coefficients increasing from 3.6$\:\pm\:$0.4 × 10⁻⁹ m²/s at 30 °C to 6.0$\:\pm\:$0.3 × 10⁻⁹ m²/s at 60 °C in distilled water.
- Additional experiments revealed that increased salinity leads to a minor reduction in hydrogen diffusion, aligning with theoretical predictions and previous molecular simulation studies.

These results have important implications for modeling hydrogen behavior in geological formations. The availability of hydrogen in the aqueous phase, governed by both diffusion and solubility, plays a key role in geochemical and microbial reactions, including those involving sulphate-reducing bacteria and pyrite, which can lead to the formation of H₂S and CH₄. Accurate characterization of hydrogen diffusion is thus essential for assessing storage efficiency, gas retention, and long-term system integrity in UHS projects.

Beyond geological storage, this methodology also provides valuable input for other hydrogen-related technologies where gas–liquid interactions are critical, such as water electrolysis, fuel cells, and hydrogen-sensing applications. Overall, this work contributes a robust and generalizable tool for quantifying hydrogen transport in aqueous systems under subsurface-relevant conditions.

## Supplementary Information

Below is the link to the electronic supplementary material.

Supplementary Material 1

## Data Availability

The authors declare that the data supporting the findings of this study are available within the paper.

## Abbreviations

A: Cross section, m^2^
b: Intercept, MPa
c: H_2_ concentration in water/brine, gmole of H_2_/m^3^ of solution
c_sat_: Maximum solubility of H_2_ in brine, gmole of H_2_/m^3^ of solution
D: H_2_ diffusion coefficient in brine, m^2^/s
$\:\bar{\mathrm{D}}$: Average diffusion coefficient, m^2^/s
erf: Error function, $\:\mathrm{erf}\left(w\right)=\frac{2}{\sqrt{\pi\:}}{\int\:}_{0}^{w}{e}^{-{t}^{2}}dt$
H: Cell height, m
j: Molar flux of H_2_ at the interface, gmole/(m^2^.s)
L: Water/brine length, m
m: Slope, MPa/$$\:\sqrt{s}$$
n: Amount of gas in the gas phase, gmole

## Greek letters

$\:{{\upsigma\:}}_{\mathrm{D}}$: Standard deviation of the diffusion coefficient, m^2^/s
n_d_: Amount of diffused H_2_ in the brine, gmole
P: Gas pressure, MPa
R: Universal gas constant, Pa.m^3^/(gmole.K)
T: Temperature, K
t: Time, s
t_0_: End of disturbance or convection mixing period, s
V_g_: Gas volume, m^3^
w: $$\:\frac{z}{2\sqrt{Dt}}$$, –
z: z-Coordinate, m
Zg: Gas compressibility factor, –
$\:{\bar{Z}}_{g}$: Average gas compressibility, –

## References

[1] Zivar, D., Kumar, S. & Foroozesh, J. Underground hydrogen storage: a comprehensive review. *Int. J. Hydrogen Energy*. **46**, 23436–23462 (2021).

[2] Wiebe, R. & Gaddy, V. L. The solubility of hydrogen in water at 0, 50, 75 and 100° from 25 to 1000 atmospheres. *J. Am. Chem. Soc.* **56**, 76–79 (1934).

[3] Chabab, S., Théveneau, P., Coquelet, C., Corvisier, J. & Paricaud, P. Measurements and predictive models of high-pressure H2 solubility in brine (H2O + NaCl) for underground hydrogen storage application. *Int. J. Hydrogen Energy*. **45**, 32206–32220 (2020).

[4] Torín-Ollarves, G. A. & Trusler, J. P. M. Solubility of hydrogen in sodium chloride brine at high pressures. *Fluid Phase Equilib.* **539**, 113025 (2021).

[5] Zhu, Z., Cao, Y., Zheng, Z. & Chen, D. An accurate model for estimating H2 solubility in pure water and aqueous NaCl solutions. *Energies (Basel)*. **15**, 5021 (2022).

[6] Ghasemi, M., Omrani, S., Mahmoodpour, S. & Zhou, T. Molecular dynamics simulation of hydrogen diffusion in water-saturated clay minerals; implications for underground hydrogen storage (UHS). *Int. J. Hydrogen Energy*. **47**, 24871–24885 (2022).

[7] Rezk, M. G., Foroozesh, J., Abdulrahman, A. & Gholinezhad, J. CO2 diffusion and dispersion in porous media: review of advances in experimental measurements and mathematical models. *Energy Fuels*. **36**, 133–155 (2022).

[8] Tsimpanogiannis, I. N., Maity, S., Celebi, A. T. & Moultos, O. A. Engineering model for predicting the intradiffusion coefficients of hydrogen and oxygen in vapor, liquid, and supercritical water based on molecular dynamics simulations. *J. Chem. Eng. Data*. **66**, 3226–3244 (2021).

[9] van Rooijen, W. A. et al. Interfacial tensions, solubilities, and transport properties of the H2/H2O/NaCl system: a molecular simulation study. *J. Chem. Eng. Data*. **69**, 307–319 (2024). 38352074 10.1021/acs.jced.2c00707 PMC10859954

[10] Wang, S., Zhou, T., Pan, Z. & Trusler, J. P. M. Diffusion Coefficients of N2O and H2 in Water at Temperatures between 298.15 and 423.15 K with Pressures up to 30 MPa. *J. Chem. Eng. Data*. **68**, 1313–1319 (2023).

[11] Riazi, M. R. A new method for experimental measurement of diffusion coefficients in reservoir fluids. *J. Pet. Sci. Eng.* **14**, 235–250 (1996).

[12] Zhang, W., Wu, S., Ren, S., Zhang, L. & Li, J. The modeling and experimental studies on the diffusion coefficient of CO2 in saline water. *J. CO2 Utilization*. **11**, 49–53 (2015).

[13] Ahmadi, H., Jamialahmadi, M., Soulgani, B. S., Dinarvand, N. & Sharafi, M. S. Experimental study and modelling on diffusion coefficient of CO2 in water. *Fluid Phase Equilib.* **523**, 112584 (2020).

[14] Wang, Z. & Hou, J. Measurement of CO2 diffusion coefficients in both bulk liquids and carven filling porous media of fractured-vuggy carbonate reservoirs at 50 MPa and 393 K. *RSC Adv.* **11**, 19712–19722 (2021). 35479232 10.1039/d1ra02549j PMC9033683

[15] Basilio, E., Addassi, M., Al-Juaied, M., Hassanizadeh, S. M. & Hoteit, H. Improved pressure decay method for measuring CO2-water diffusion coefficient without convection interference. *Adv. Water Resour.* **183**, 104608 (2024).

[16] Khajooie, S. et al. Exploring effective diffusion coefficients in Water-Saturated reservoir rocks via the pressure decay technique: implications for underground hydrogen storage. *Transp. Porous Media*. **152**, 12 (2025).

[17] Tsimpanogiannis, I. N. & Moultos, O. A. Is stokes-einstein relation valid for the description of intra-diffusivity of hydrogen and oxygen in liquid water? *Fluid Phase Equilib.* **563**, 113568 (2023).

[18] Rezk, M. G. Measurement of hydrogen diffusion in brine at high pressures and elevated temperatures: implications for underground hydrogen storage. *Int. J. Hydrogen Energy*. **139**, 496–509 (2025).

[19] Paul, A., Laurila, T., Vuorinen, V. & Divinski, S. V. Fick’s laws of diffusion. in *Thermodynamics, Diffusion and the Kirkendall Effect in Solids* 115–139 (Springer International Publishing, Cham). 10.1007/978-3-319-07461-0_3 (2014).

[20] Zhang, Y. et al. Experimental measurements of the diffusion coefficient and effective diffusion coefficient of CO2–Brine under offshore CO2 storage conditions. *Energy Fuels*. **37**, 19695–19703 (2023).

[21] Maharajh, D. M. & Walkley, J. The temperature dependence of the diffusion coefficients of Ar, CO2, CH4, CH3Cl, CH3Br, and CHCl2F in water. *Can. J. Chem.* **51**, 944–952 (1973).

[22] Zarghami, S., Boukadi, F. & Al-Wahaibi, Y. Diffusion of carbon dioxide in formation water as a result of CO2 enhanced oil recovery and CO2 sequestration. *J. Pet. Explor. Prod. Technol.* **7**, 161–168 (2017).

[23] Frank, M. J. W., Kuipers, J. A. M. & van Swaaij, W. P. M. Diffusion coefficients and viscosities of CO2 + H2O, CO2 + CH3OH, NH3 + H2O, and NH3 + CH3OH liquid mixtures. *J. Chem. Eng. Data*. **41**, 297–302 (1996).

[24] Li, Z., Yuan, L., Sun, G., Lv, J. & Zhang, Y. Experimental determination of CO2 diffusion coefficient in a Brine-Saturated core simulating reservoir condition. *Energies (Basel)*. **14**, 540 (2021).

[25] Azin, R., Mahmoudy, M., Raad, S. & Osfouri, S. Measurement and modeling of CO2 diffusion coefficient in saline aquifer at reservoir conditions. *Cent. Eur. J. Eng.* **3**(4), 585–594 (2013).

[26] Cadogan, S. P., Maitland, G. C. & Trusler, J. P. M. Diffusion Coefficients of CO2 and N2 in Water at Temperatures between 298.15 K and 423.15 K at Pressures up to 45 MPa. *J. Chem. Eng. Data*. **59**, 519–525 (2014).

[27] Sharafi, M. S., Ghasemi, M., Ahmadi, M. & Kazemi, A. An experimental approach for measuring carbon dioxide diffusion coefficient in water and oil under supercritical conditions. *Chin. J. Chem. Eng.* **34**, 160–170 (2021).

[28] Lu, W., Guo, H., Chou, I. M., Burruss, R. C. & Li, L. Determination of diffusion coefficients of carbon dioxide in water between 268 and 473 K in a high-pressure capillary optical cell with in situ Raman spectroscopic measurements. *Geochim. Cosmochim. Acta*. **115**, 183–204 (2013).

[29] Wise, D. L. & Houghton, G. The diffusion coefficients of ten slightly soluble gases in water at 10–60°C. *Chem. Eng. Sci.* **21**, 999–1010 (1966).

[30] Ferrell, R. T. & Himmelblau, D. M. Diffusion coefficients of hydrogen and helium in water. *AIChE J.* **13**, 702–708 (1967).

[31] de Blok, W. J. & Fortuin, J. M. H. Method for determining diffusion coefficients of slightly soluble gases in liquids. *Chem. Eng. Sci.* **36**, 1687–1694 (1981).

[32] Jähne, B., Heinz, G. & Dietrich, W. Measurement of the diffusion coefficients of sparingly soluble gases in water. *J. Geophys. Res. Oceans*. **92**, 10767–10776 (1987).

[33] Kalati, S. S. et al. Molecular dynamics simulation of hydrogen diffusion into brine: implications for underground hydrogen storage. *Int. J. Hydrogen Energy*. **53**, 17–28 (2024).

[34] Kerkache, H. et al. Assessment of H2 diffusivity in water and Brine for underground storage: a molecular dynamics approach. *Int. J. Hydrogen Energy*. **128**, 279–290 (2025).

[35] Bhimineni, S. H. et al. Machine-learning-assisted investigation of the diffusion of hydrogen in brine by performing molecular dynamics simulation. *Ind. Eng. Chem. Res.* **62**, 21385–21396 (2023).

[36] Piroozi, G., Kouhi, M. M. & Shafiei, A. Novel intelligent models for prediction of hydrogen diffusion coefficient in brine using experimental and molecular dynamics simulation data: implications for underground hydrogen storage in geological formations. *J. Energy Storage*. **118**, 116297 (2025).

[37] Etminan, S. R., Maini, B. B., Chen, Z. & Hassanzadeh, H. Constant-pressure technique for gas diffusivity and solubility measurements in heavy oil and bitumen. *Energy Fuels*. **24**, 533–549 (2010).

[38] Kolev, N. I. Solubility of O2, N2, H2 and CO2 in water. in *Multiphase Flow Dynamics 4* 209–239 (Springer Berlin Heidelberg, Berlin, Heidelberg, 2011). 10.1007/978-3-642-20749-5_11

[39] Poling, B. E., Prausnitz, J. M. & O’Connell, J. P. *The Properties of Gases and Liquids*, 5th Edition. McGraw-Hill: New York (2001).

