# Supplementary material for: An experimental study and mathematical formulation for hydrogen diffusion in water
Source: Sci Rep. 2025 Dec 29;15:44790. doi: 10.1038/s41598-025-28427-2 (PMC12749415; doi:10.1038/s41598-025-28427-2)
Supplement: Supplementary file 1 — Supplementary Material 1 [file 41598_2025_28427_MOESM1_ESM.pdf]

1) Diffusion of CO_2 in distilled water

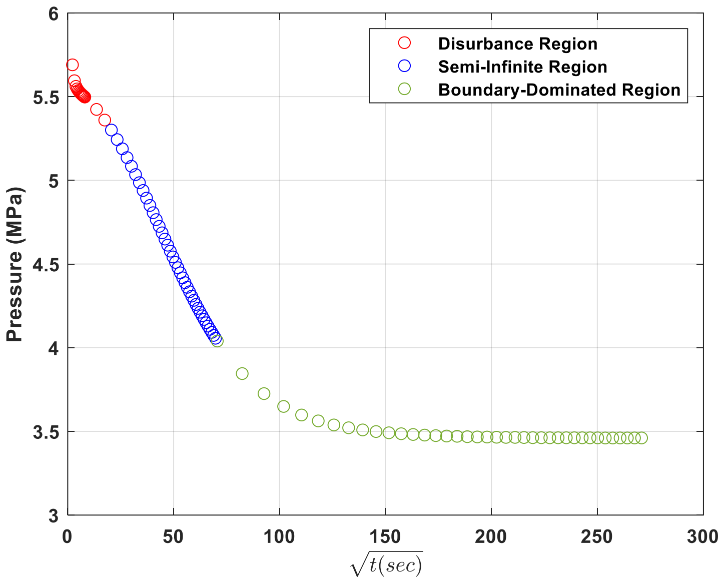

Figure S1: Pressure profile versus the square root of time for Case 1

2) Diffusion of H_2 in distilled water

(a)

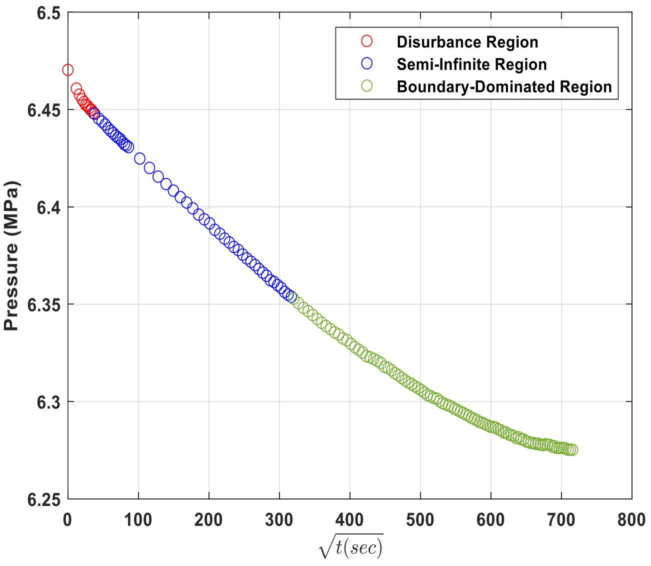

(b)

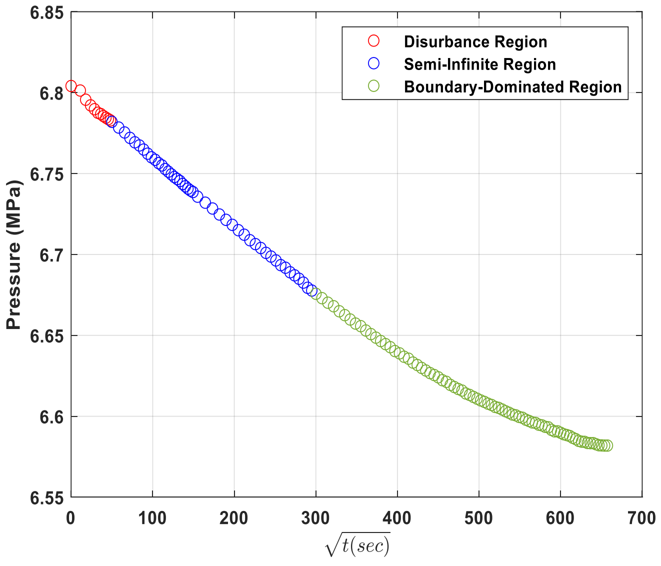

(c)

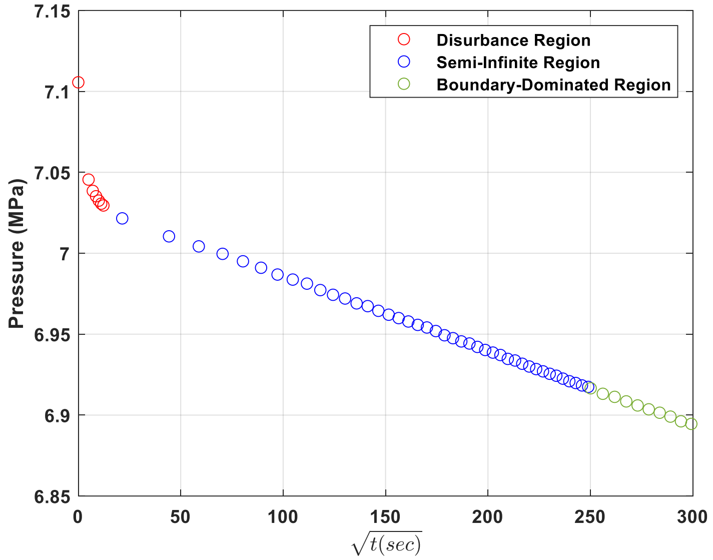

Figure S2: Pressure profile versus square root of time for Case 1a (a), Case 2a (b), Case 3b (c).

3) Diffusion of H_2 in brine

(a)

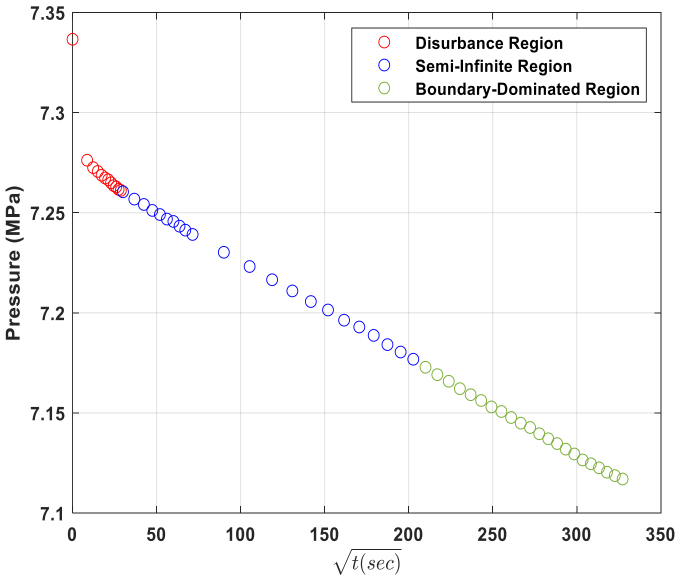

(b)

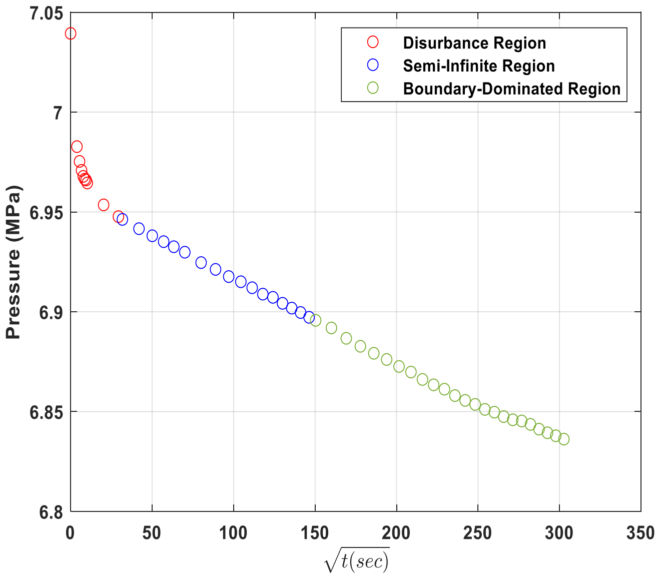

Figure S3: Pressure profile versus square root of time for Case 1b (a), Case 2a (b).
